# A Bionic Goal-Oriented Path Planning Method Based on an Experience Map

**DOI:** 10.3390/biomimetics10050305

**Published:** 2025-05-11

**Authors:** Qiang Zou, Yiwei Chen

**Affiliations:** 1 Faculty of Robot Science and Engineering, Northeastern University, Shenyang 110819, China; yiweichen@stumail.neu.edu.cn; 2 Foshan Graduate School of Innovation, Northeastern University, Foshan 528311, China

**Keywords:** bionic goal-oriented path planning, experience map, successor representation model

## Abstract

Brain-inspired bionic navigation is a groundbreaking technological approach that emulates the biological navigation systems found in mammalian brains. This innovative method leverages experiences within cognitive space to plan global paths to targets, showcasing remarkable autonomy and adaptability to various environments. This work introduces a novel bionic, goal-oriented path planning approach for mobile robots. First, an experience map is constructed using NeuroSLAM, a bio-inspired simultaneous localization and mapping method. Based on this experience map, a successor representation model is then developed through reinforcement learning, and a goal-oriented predictive map is formulated to address long-term reward estimation challenges. By integrating goal-oriented rewards, the proposed algorithm efficiently plans optimal global paths in complex environments for mobile robots. Our experimental validation demonstrates the method’s effectiveness in experience sequence prediction and goal-oriented global path planning. The comparative results highlight its superior performance over traditional Dijkstra’s algorithm, particularly in terms of adaptability to environmental changes and computational efficiency in optimal global path generation.

## 1. Introduction

Autonomous navigation is a critical capability for mobile robots operating in unstructured and unfamiliar environments [1]. Conventional navigation approaches primarily focus on precise geometric parameter estimation, often involving incremental enhancements to established algorithms [2]. However, these methods often lack the cognitive mechanisms to process perceptual data into meaningful memories or adaptive knowledge. Consequently, their performance in complex scenarios is constrained by limited robustness and insufficient cognitive flexibility [3], which hinders their ability to support truly intelligent autonomous operation in demanding real-world applications [4,5]. In contrast to artificial navigation systems, mammals in natural environments can perform sophisticated spatial tasks without relying on precise metric measurements. This ability arises from the intrinsic spatial processing mechanisms of their brains, which allow for robust environmental recognition and self-localization, even in unfamiliar settings [6]. Inspired by this biological principle, brain-inspired bionic navigation has emerged as a novel paradigm that replicates neural spatial computation. Unlike traditional methods, this approach utilizes learned environmental representations in cognitive space to dynamically guide movement toward goals, demonstrating enhanced autonomy and adaptive flexibility. Such biomimetic strategies may provide mobile robots with a more efficient solution for achieving precise positioning and rapid navigation in complex real-world scenarios [7].

Recently, some neuroscientists discovered that the place cells [8] in the hippocampus and grid cells [9] in the entorhinal cortex are essential nerve cells involved in memory, cognitive mapping, and navigation [10,11]. These neural ensembles encode integrated environmental representations that encompass both memory-based and metric spatial frameworks [12]. Through memory recall processes, mammals can infer current and anticipated spatial states, enabling rapid path planning while enhancing situational awareness and behavioral flexibility in dynamic environments [13,14,15]. This neurocognitive framework has inspired considerable research in bio-inspired navigation, leading to the development of several computational models that emulate mammalian spatial intelligence. Among these models, various bionic methods have been proposed for map construction. Milford et al. [16,17] proposed the biologically inspired RatSLAM algorithm for constructing experience maps in two-dimentsional space. Building on RatSLAM, Yu et al. [18] introduced NeuroSLAM, which enabled 3D experience map construction. Tejera et al. [19] developed a neuromorphic spatial cognition model capable of generating topological world graphs for robotic navigation systems. Several studies have focused primarily on bionic navigation. Milford et al. [20] constructed environmental experience maps using RatSLAM and derived optimal navigation paths through gradient optimization techniques. Stachowicz et al. [21] introduced an indexed data structure for experience storage in robotic systems. Naghizadeh et al. [22] established a grid cell-based computational framework with dynamic weight adjustment capabilities to support navigation decisions. Zou et al. [23,24] introduced a global path planning methodology based on cognitive maps, enabling robots to reconstruct experience node sequences and anticipate optimal navigation paths to their targets. Additionally, several bionic navigation methods based on reinforcement learning (RL) have been proposed. Banino et al. [25] developed a deep learning framework that computationally emulates grid cell-mediated path integration, allowing trained artificial agents to perform vector-based navigation in simulated environments. Scleidorovich et al. [26,27] established a hippocampal-inspired reinforcement learning architecture that leverages multi-scale spatial representations along the dorsal–ventral axis. Their findings demonstrate that smaller-scale representations are crucial for enhancing path planning, while larger-scale representations are beneficial for reducing learning time and reducing the number of cells required. Yu et al. [28] developed a bio-inspired navigation framework that employs spiking neural networks to encode continuous state-action spaces through computational models of place cells and action cells. This architecture, combined with direct reinforcement learning, facilitates target-driven autonomous navigation. Yuan et al. [29] implemented a bio-inspired navigation system that integrates place cell localization, grid cell path integration, and boundary cell detection mechanisms, enabling efficient goal-directed navigation in simulated environments. Building upon the RatSLAM architecture, Yu et al. [30] developed a biologically inspired path planning framework that provides robotic systems with enhanced flexibility and intelligence in navigation decision-making. The aforementioned bionic navigation methods primarily focus on either establishing functional models to examine the neural mechanisms of spatial cognition or developing efficient goal-directed strategies for small-scale simulated environments. These RL-based navigation methods typically require extensive training in various environments to discover optimal paths to destination points, they often lack sufficient experience-based planning and are not suitable for robotic applications in real environments.

The predictive map framework [31] was introduced to account for place cell response characteristics beyond conventional cognitive map interpretations. This model generates future state predictions based on current states, enabling long-term reward forecasting and aligning with extensive findings from hippocampal experiments. Currently, researchers have integrated the predictive map into robotic path planning algorithms. Empirical evaluations demonstrate the theoretical validity and practical efficacy of this approach for developing navigation algorithms. Inspired by this bionic concept, this work constructs a predictive map based on experience map. This predictive map is then utilized to tackle the challenge of goal-oriented path planning, enabling the anticipation and reorganization of experience sequences within complex environments. The main contributions of this research are summarized as follows:

1. A bionic goal-oriented global path planning framework has been developed to address the challenge of rapid global path planning in complex environments;
2. An experience map-based successor representation model is constructed, and it is integrated with reinforcement learning to create the predictive map, addressing the challenge of long-term reward prediction. This approach facilitates the rapid planning of the experience sequence towards the navigation goal;
3. Compared to the traditional path planning method, the proposed method demonstrates enhanced adaptability to environmental changes, and plans the optimal path with high efficiency.

The remainder of this paper is organized as follows. Section 2 presents the overall framework of the proposed method. Section 3 elaborates on the global path planning method. Subsequently, Section 4 details a series of experiments, analyzing and discussing the experimental results. Finally, Section 5 concludes the paper.

## 2. System Overview

**Figure 1:**
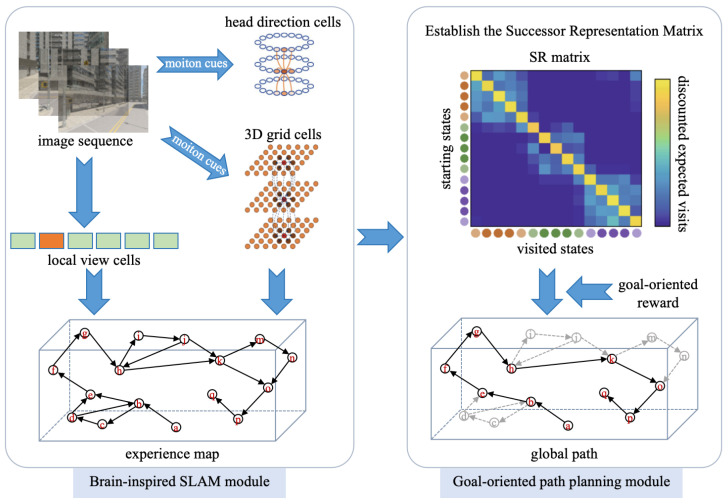
System framework.

The overall framework of the proposed system is illustrated in Figure 1; it consists of two key modules, the brain-inspired SLAM module and the goal-oriented path planning module, respectively. As the robot navigates its environment, its vision sensor continuously captures scene images, generating a time-series image sequence. This image sequence is input into the brain-inspired SLAM module, which processes the images to construct an experience map. The generated experience map is then fed into the goal-oriented path planning module. By building the successor representation matrix and defining the goal-oriented reward vector, the system can anticipate an optimal global path to the specified target.

NeuroSLAM is a state-of-the-art, neurally inspired SLAM framework that bridges the gap between biological neural processes and artificial intelligence for navigation. It leverages the latest insights into neural coding and processing in the mammalian brain, particularly regarding spatial awareness and navigation. The NeuroSLAM architecture integrates three core components: (1) a conjunctive pose cell network fusing 3D grid cell representations with multi-layered head-directional coding, (2) a spatial experience mapping module, and (3) visual processing units. The system derives local view descriptors and self-motion estimates from sequential images captured by the camera, with the pose cell network executing path integration through the coordinated processing of visual and motion cues. Distinct scenes are encoded by the local view cells. The integration of local view cell activations, 3D grid cell outputs, multi-layered head-direction signals, and visual odometry data generates a three-dimensional experiential map, which serves as a hybrid spatial representation that combines topological relationships with local metric precision. Each 3D spatial experience is characterized by unique activation patterns in both local view cells and conjunctive pose cells. Alterations in these patterns trigger the formation of new spatial experiences, accompanied by corresponding transitions that encode 4DoF pose variations. During environmental exploration, this system continuously generates new experiences and inter-experience transitions. When familiar surroundings are re-encountered, loop closure is triggered when conjunctive codes match stored representations, with subsequent map relaxation maintaining topological coherence.

The goal-oriented global path planning module is the primary contribution in this work. It employs a successor representation framework that encodes states based on their predicted future state distributions. Using the constructed experience map, a successor representation matrix is established through reinforcement learning, enabling the rapid construction of a goal-oriented predictive map for anticipating an optimal global path to the navigation goal quickly. A detailed description of this global path planning module will be provided in the next section. It is important to note that the focus of this research does not include how mobile robots perform local path planning and motion control based on the planned global path.

## 3. Global Path Planning

Effective long-term reward prediction is a crucial cognitive capability for mammalian survival. Mammals can experience intervals spanning days, weeks, or even months before achieving a primary reward. Evidence suggests that the brain has developed various of strategies to tackle the reinforcement learning problem. These strategies are typically classified into the following categories:

- The first strategy involves using learned environmental representations to simulate potential state sequences for reward prediction. However, this strategy presents significant computational challenges in real-world environments due to the exponential growth of possible future states.
- The second strategy relies on trial-and-error learning to approximate a value function that predicts future rewards based on current states. However, this approach struggles in dynamic environments, where evolving reward contingencies require continuous re-optimization of value estimates.
- The third strategy utilizes a successor representation framework that encodes states based on their predicted future state distributions. This representation allows for efficient long-term reward estimation while remaining computationally tractable and neurobiologically plausible, aligning closely with empirical observations of hippocampal function.

According to the predictive map paradigm, place cells encode probabilistic future state distributions rather than static positional information. When integrated with reward prediction mechanisms, this representation facilitates value computation, offering a unified explanation for how environmental variables (such as obstacles, spatial layout, and movement direction) modulate firing patterns. This theoretical framework also extends naturally to the hippocampal involvement in non-spatial cognitive tasks.

### 3.1. Predictive Map Construction

To construct the predictive map, this work integrates the experience map with reinforcement learning, and assumes that the predictive map construction process conforms to the Markov Decision Process. Let *s* represent the robotic state, which is considered as the experience during environmental exploration, and *S* denote the set of experiences. $P(s^{\prime }|s,C)$ denotes the transition probability from state *s* to state $s^{\prime }$ under the connection relationship *C*. R(s) is the obtained reward in state *s*. In this work, the reward is computed based on edge traversal counts between the current experience vertex and its adjacent vertices in the topological graph. A discount factor γ is defined to reduce the forward reward, γ∈[0,1]. During the learning phase, the robotic system first selects transition states based on the policy π(C|s) and then acquires the associated reinforcement values. The state-value function V(s) represents the expected cumulative discounted return, as Equation (1):

(1) $$V\left(s\right)=E_{\pi }\left[\sum_{t=0}^{N}\gamma^{t}R\left(s_{t}\right)|s_{0}=s\right],$$

where *N* defines the number of experience nodes within the environmental experience map, and $s_{t}$ represents the agent’s state at timestep *t*.

Computationally, the state-value function can be separated into predictive coding $T(s,s^{\prime })$ and the reward function, as shown in Equation (2):

(2) $$V\left(s\right)=\sum_{s^{\prime }}T\left(s,s^{\prime }\right)R\left(s^{\prime }\right),$$

where $T(s,s^{\prime })$ is known as successor representation (SR), denoting the discounted future state occupancy measurement, as shown in Equation (3):

(3) $$T\left(s,s^{\prime }\right)=E\left[\sum_{t=0}^{N}f\left(s_{t}=s^{\prime }\right)|s_{0}=s\right],$$

where *f* is the indicator function if $s_{t}=s^{\prime }$, $f(s_{t}=s^{\prime })=1$; otherwise, $f(s_{t}=s^{\prime })=0$. In this manner, the expected cumulative reward can be expressed as the inner product of the discounted state occupancy matrix and the reward vector.

As a fundamental algorithm in reinforcement learning, Temporal Difference (TD) learning combines principles from dynamic programming with sampling techniques characteristic of Monte Carlo methods. This approach enables an agent to learn from the environment by computing state-action value function through the iterative minimization of temporal prediction errors. In this work, the estimate of the SR is updated incrementally using the TD learning algorithm. $\hat{T}$ is updated through Equation (4):

(4) $${\hat{T}}_{t+1}\left(s_{t},s^{\prime }\right)={\hat{T}}_{t}\left(s_{t},s^{\prime }\right)+\eta \left[f\left(s_{t}=s^{\prime }\right)+\gamma {\hat{T}}_{t}\left(s_{t+1},s^{\prime }\right)-{\hat{T}}_{t}\left(s_{t},s^{\prime }\right)\right],$$

where η is the learning rate. In this work, we choose a common and well-justified parameter combination for the discount factor and learning rate, i.e., γ = 0.9 and η = 0.1. The selection of a discount factor aims to balance medium-term reward capture while avoiding excessive focus on distant rewards in partially observable environments, and the selection of learning rate aims to balance update stability with convergence speed.

**Figure 2:**
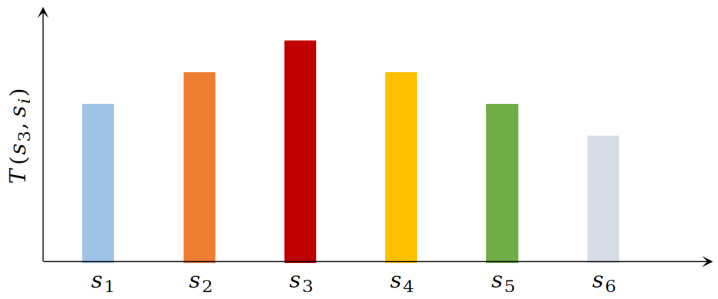
Schematic diagram of SR value.

In general, reinforcement learning-based goal-oriented navigation methods typically begin from a given starting position and gradually explore the environment to approach the target position. However, when the environment is dynamic or the target is changing, these methods require a modulation of the reward function and retraining. Specifically, the value function in this work is decomposed into the state’s connection SR matrix and the reward function; the reward, independent of the SR matrix, allows for the rapid calculation of a new reward function. Figure 2 illustrates the schematic diagram of the SR value. Here, $T(s_{3},s)$ denotes the connection relationship between state $s_{3}$ and state *s* at time *t*. This work defines the connection relationship as the expected visit times from state $s_{3}$ to state *s*, represented as one column vector in SR matrix. In Figure 2, since the agent is currently in state $s_{3}$, the SR values in adjacent states ($s_{2}$ and $s_{4}$) are higher, indicating a stronger connection between state $s_{3}$ and its adjacent states. Consequently, the visit times to these adjacent states should be greater than those to non-adjacent states.

### 3.2. Convergence Rule of SR Model

To ensure that the mobile robot can establish a complete global connection among a large number of experience nodes, it is essential to train the SR model adequately. Let $p_{ik}\left(t\right)$ represent the probability of transitioning to the next optional connection state *k* from the *i*th state at timestep *t*, and $p_{ik}\left(t+1\right)$ denote the probability for the same transition at timestep t+1. If the value of the current state increases after selecting the next state *k*, then the probability of selecting state *k* should be increased accordingly, as shown in Equation (5):

(5) $$p_{ik}\left(t+1\right)=p_{ik}\left(t\right)+a_{1}\Delta T\left(1-p_{ik}\left(t\right)\right),$$

where $a_{1}$ is a constant between 0 and 1, and ΔT represents the difference in state *k* during the training process of the SR model. Assuming that the robot is located at the *i*th state at the current time, it has three optional states at the next time, so the sum of the probabilities of connecting from the current state to the next state should be 1, as shown in Equation (6):

(6) $$\sum_{j=1}^{n}p_{ik_{j}}\left(k_{j}\left(t\right)|s_{i}\left(t\right)\right)=1,$$

where *j* is the index of the next optional state, and *n* is the number of available connection states corresponding to the current state.

Correspondingly, the probabilities of connections to other states also change, as shown in Equation (7):

(7) $$p_{iu}\left(t+1\right)=p_{iu}\left(t\right)-a_{1}\Delta T\left(1-p_{ik}\left(t\right)\right)\delta ,$$

where *u* ≠ $k_{j}$(*j*∈*n*) represents other optional connection states excluding the current connection, $\delta =p_{iu}\left(t\right)/\sum_{u=1,u\neq k}^{n}p_{iu}\left(t\right)$.

If the value of the current state decreases after selecting the next state *k*, then the probability of selecting state *k* should be reduced too, as shown in Equation (8):

(8) $$p_{ik}\left(t+1\right)=p_{ik}\left(t\right)+a_{2}\Delta Tp_{ik}\left(t\right),$$

where $a_{2}$ is also a constant between 0 and 1. The same as Equation (8), the probabilities of the connections to other states change, as shown in Equation (9):

(9) $$p_{iu}\left(t+1\right)=p_{iu}\left(t\right)-a_{2}\Delta Tp_{ik}\left(t\right)\delta ,$$

In dynamic environments, there may be an obstacle that blocks the connection between two states. If the robot encounters an obstacle while moving between these connected states, the current connection action should be set to zero. Additionally, the connection direction from the previous state should be evenly distributed among the other available connections.

Information entropy is generally equivalent to the inherent uncertainty of a variable. As the agent gains a thorough understanding of the environment and develops a clearer state-action policy through continuous exploration, the uncertainty of subsequent actions will decrease. If learning in a system reflects a reduction in uncertainty, it should also result in a decrease in the agent’s perceived information entropy. In this work, we utilize information entropy as a convergence metric to assess the stabilization of the successor representation model during training. If the information entropy value tends toward stability, the SR model is considered converged, indicating a greater degree of self-organization and a reliability of the system. The convergence information entropy is computed as Equation (10):

(10) $$H\left(t\right)=-\frac{1}{N_{s}}\sum_{i=1}^{N_{s}}\sum_{j=1}^{n}p_{ik_{j}}\left(t\right)\log p_{ik_{j}}\left(t\right),$$

where $N_{s}$ denotes the total number of states within the experience map, and *n* represents the number of available connection states for each state. When the information entropy no longer undergoes significant changes, the SR model is considered to have converged, resulting in the identification of stable connection states for each experience node.

In summary, the algorithm for constructing the SR matrix is presented in Table 1.

### 3.3. Goal-Oriented Path Planning

As shown in Equation (2), the state-value function can be expressed as the inner product of the SR matrix and the the reward vector. To enable mobile robots to perform goal-oriented global path planning, the reward vector should be defined as a function related to the target. In this work, the reward function r(s) is defined as Equation (11):

(11) $$r\left(s\right)=\left\{\begin{array}{cc} r_{+} & \mathrm{if}\;s=s_{\mathrm{target}}\\ 0 & \mathrm{otherwise} \end{array}\right.$$

where $r_{+}$ denotes the target-state reward: it is a positive reward. Given the target-related reward vector and the converged SR matrix, the mobile robot navigates toward the target by sequentially selecting the highest-value state connections, forming an optimal experience sequence.

Like biological navigation systems, the goal-oriented global path planning system demonstrates dynamic adaptation. When the robot reaches a state where its highest-value successor state is unreachable, the system will sever this topological link, and trigger replanning from the present state. In this way, the proposed goal-oriented path planning method can adapt to environmental changes.

## 4. Experimental Results

To verify our proposed methods, some experiments were implemented based on the SynPerData and QUTCarparkData datasets, respectively. Furthermore, a maze environment is constructed to evaluate the robustness of our methods in a dynamic unstructured environment. In both dataset experiments, the experience map building algorithm was implemented in MATLAB (https://www.mathworks.com/products/matlab.html (accessed on 9 April 2025)), while the goal-oriented global path planning algorithm was executed in Python. The maze experiment was fully implemented in Python 3.13.2. The computer used for these experiments is equipped with a 13th Intel Core i5 2.6 GHz CPU (Intel 2200 Mission College Blvd. Santa Clara, CA, USA). All parameters for the global path planning were kept consistent, including a discount factor γ = 0.9, a learning rate η = 0.1, and a target-state reward $r_{+}$ = 10.

### 4.1. Results Based on SynPerData

**Figure 3:**
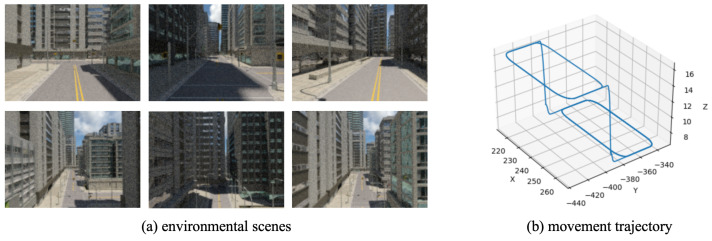
The environmental scenes and movement trajectory about SynPerData.

As a synthetic dataset, SynPerData is generated within a 3D urban environment featuring diverse architectural structures, road networks, and transportation infrastructure. The environmental scenes are depicted in Figure 3a. SynPerData comprises 4994 consecutive scene images. Figure 3b illustrates the groundtruth of the movement trajectory, clearly indicating that the camera’s trajectory is three dimensional.

**Figure 4:**
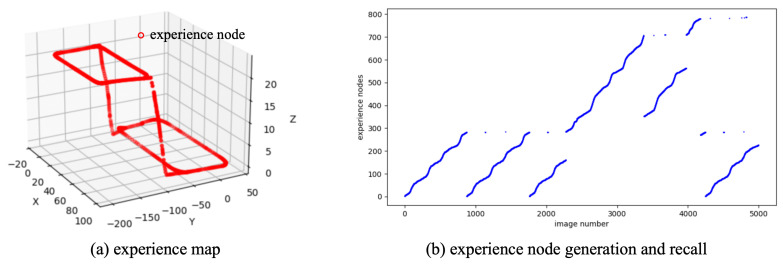
The experience map building results based on SynPerData.

Initially, the 4994 consecutive scene images are input into the NeuroSLAM system, which derives the local view descriptors and self-motion estimates from sequential images. The pose cell network performs path integration through the coordinated processing of visual and motion cues. As scene images are continuously input, the NeuroSLAM system incrementally generates new experiences and inter-experience transitions. Figure 4a displays the results of the experience map built from SynPerData, showing that the topology of the experience map aligns with the ground truth of the movement trajectory. Figure 4b illustrates the generation and recall of experience nodes along the scene images, indicating that the constructed experience map contains a total of 786 experience nodes.

**Figure 5:**
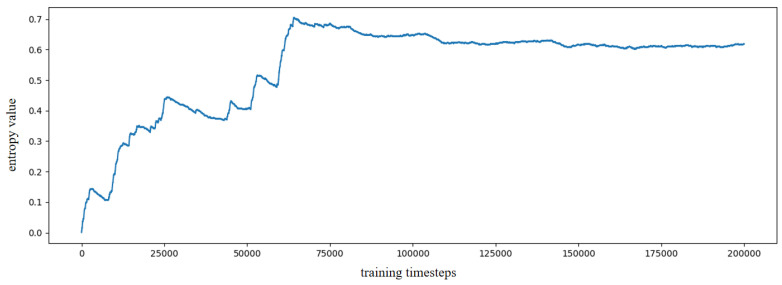
The information entropy along with training timestep based on SynPerData.

**Figure 6:**
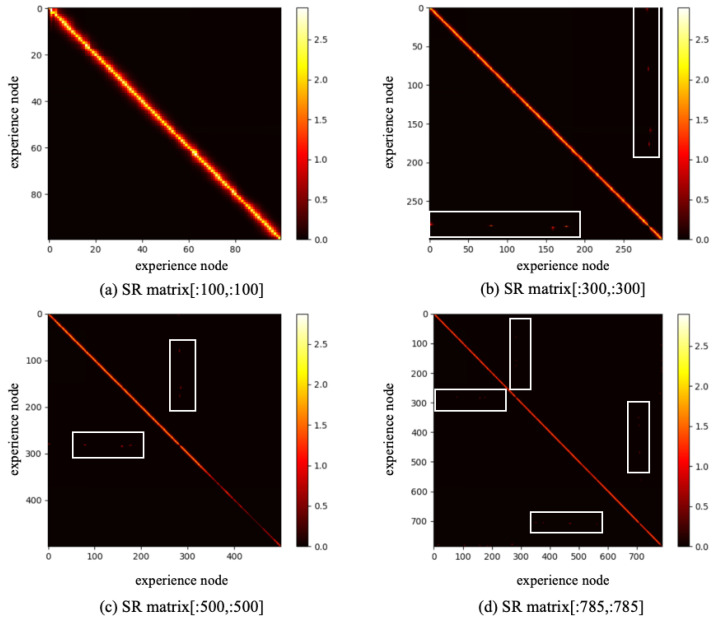
The SR matrix result based on SynPerData.

Based on the constructed experience map, several goal-oriented global path planning experiments were conducted. First, the adjacent selectable state nodes for each state node were extracted from the experience map, and the SR model was trained through reinforcement learning. During the training process, the information entropy is updated along with the training timestep. We have trained the SR model for 200,000 timesteps, and the change in information entropy is illustrated in Figure 5. It is evident that after training 100,000 timesteps, the information entropy stabilizes, indicating that the SR model has converged. Figure 6 presents the converged SR matrix, which details the transition weights among these experience nodes. Specifically, Figure 6a shows the heatmap of the SR matrix for the first 100 experience nodes, while Figure 6b,c displays the heatmaps for the first 300 and first 500 experience nodes, respectively. Figure 6d provides the heatmap for all experience nodes. In the figures, the white rectangle box indicates that there are interconnected experience nodes in that area.

**Figure 7:**
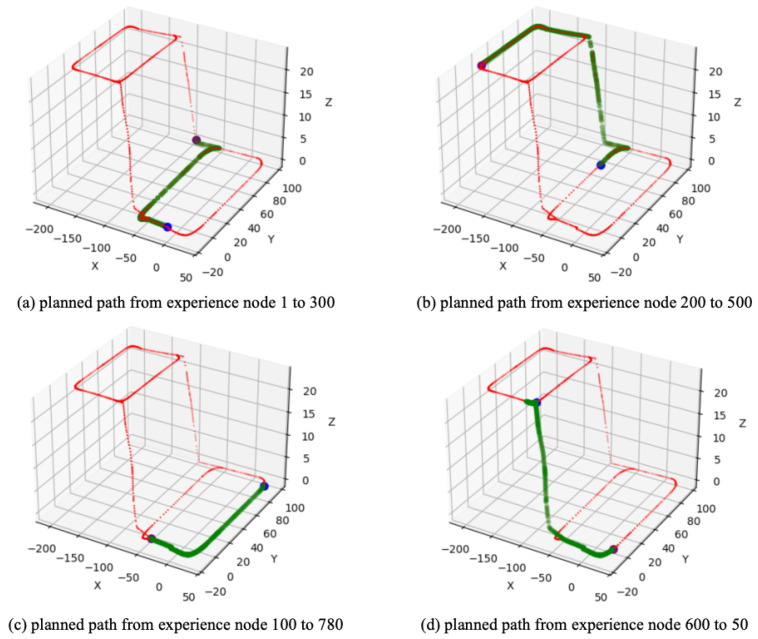
The global path planning result based on SynPerData. The red dots represent the experience map, the blue dot represents the start point, the purple dot represents the goal point, and the green dots represent the planned experience nodes sequence.

After the SR matrix is converged, different goal-oriented global path planning experiments are carried out. Figure 7a shows the planned path from experience node 1 (start point) to experience node 300 (goal point). It is clear that the planned path differs from the learning path; specifically, the planned path is a subset of the learning path. Similarly, Figure 7b,c present the results of global path planning from experience node 200 to 500 and from experience node 100 to 700, respectively. The first three experiments belong to forward planning, which are different from these; Figure 7d shows the planned path from experience node 600 to 50, demonstrating that our method is also effective for reverse planning.

To validate the high robustness and efficiency of our method, the Dijkstra method was adopted to complete the same four path planning experiments, and the time cost is recorded in Table 2. The comparison results show that our method can robustly generate the same global path as Dijkstra method. Moreover, our method demonstrates superior computational efficiency compared to the Dijkstra method. In conclusion, our method not only effectively plans an optimal global path to the goal but also achieves higher planning efficiency.

### 4.2. Results Based on QUTCarparkData

**Figure 8:**
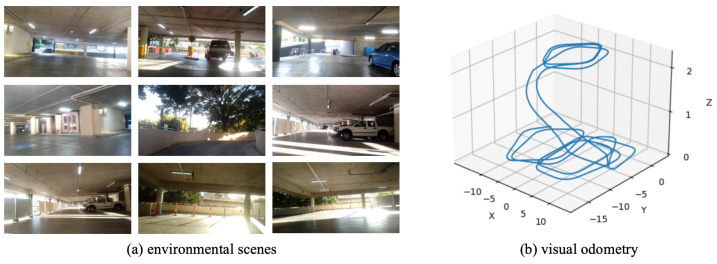
The environmental scenes and movement trajectory of QUTCarparkData.

**Figure 9:**
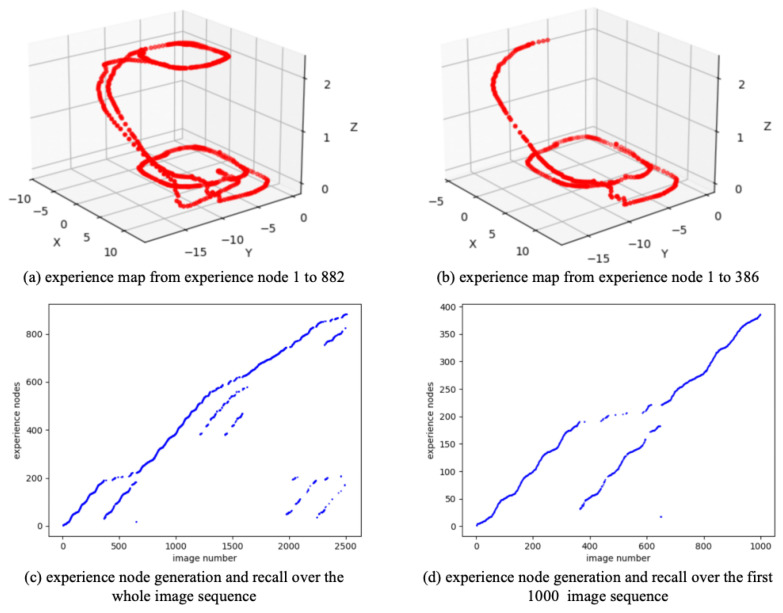
The experience map building results based on QUTCarparkData.

As a real-world dataset, QUTCarparkData is collected from a two-level parking facility at Queensland University of Technology, encompassing both indoor garage structures and outdoor parking areas. Figure 8 shows the scenes and trajectory of QUTCarparkData. QUTCarparkData consists of 2510 consecutive scene images. Utilizing the NeuroSLAM method, an experience map is constructed. As shown in Figure 9, Figure 9a shows the experience map building result, and Figure 9c describes the generation and recall of experience nodes along the scene images; it generates 882 experience nodes in total.

**Figure 10:**
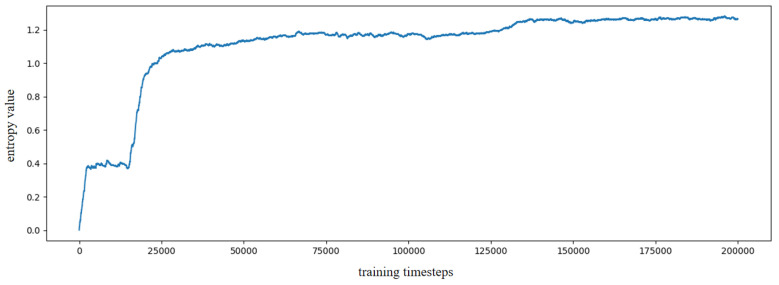
The information entropy along with the training timestep based on QUTCarparkData.

**Figure 11:**
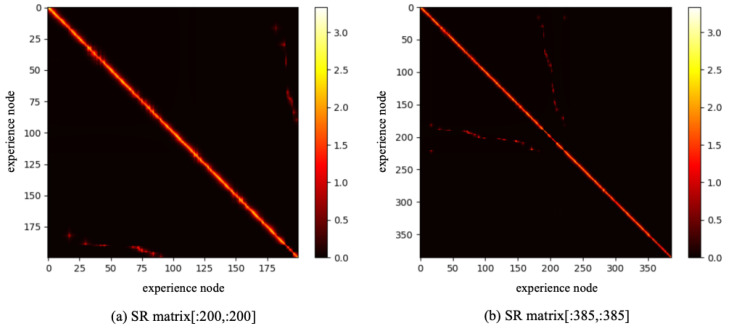
The SR matrix result based on QUTCarparkData.

To reduce the training burden of the SR model, this work extracts the first 1000 image sequence first, and the extracted experience map consists of 386 experience nodes, as illustrated in Figure 9b. The SR model is then trained for 200,000 timesteps, with the change in information entropy represented in Figure 10. It is evident that after 30,000 timesteps, the information entropy stabilizes, indicating that the SR model has converged. The converged SR matrix is displayed in Figure 11. Figure 11a presents the heatmap of the SR matrix for the first 200 experience nodes, while Figure 11b shows the heatmap for all experience nodes.

**Figure 12:**
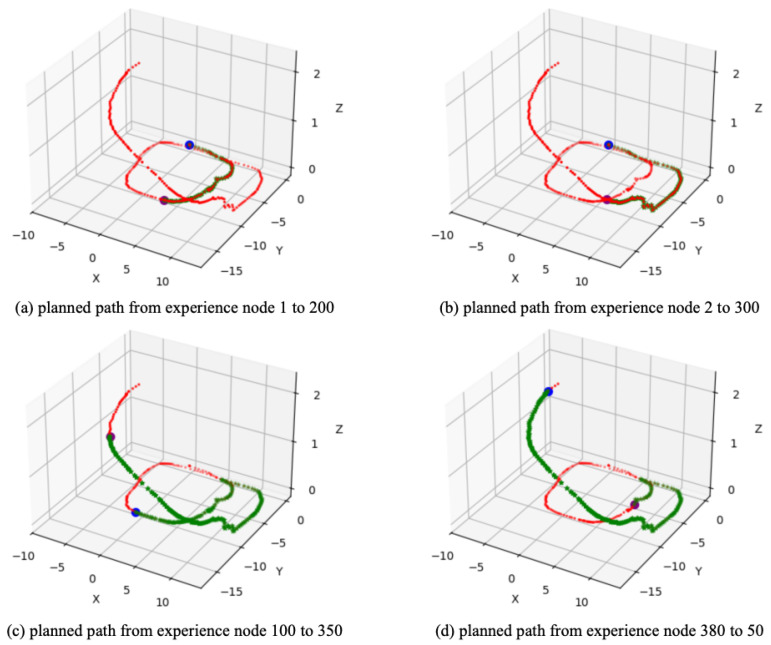
The global path planning result based on QUTCarparkData. The red dots represent the experience map, the blue dot represents the start point, the purple dot represents the goal point, and the green dots represent the planned experience nodes sequence.

Based on the converged SR matrix, four path planning experiments were conducted. Figure 12a shows the planned path from experience node 1 (start point) to experience node 200 (goal point); similarly, Figure 12b,c show the results of global path planning from experience node 2 to 300 and from experience node 100 to 350, respectively, while Figure 12d shows the planned path from experience node 380 to 50. The goal-oriented path planning results clearly demonstrate that our method effectively plans an optimal global path to the goal, whether in forward or reverse planning. Additionally, Table 3 compares the time costs between our method and the Dijkstra method, further validating that our approach offers higher planning efficiency.

### 4.3. Results in Maze Environment

**Figure 13:**
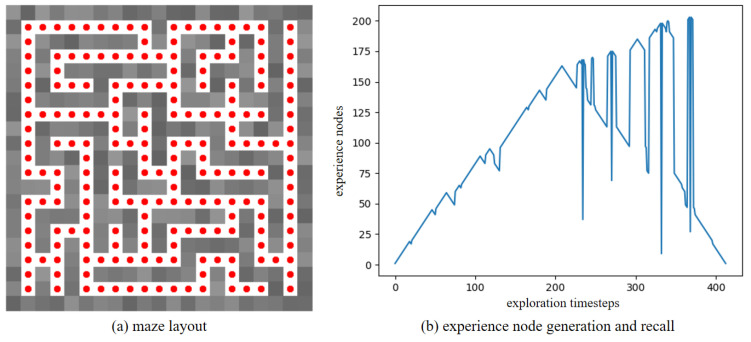
The exploration results for the maze environment.

**Figure 14:**
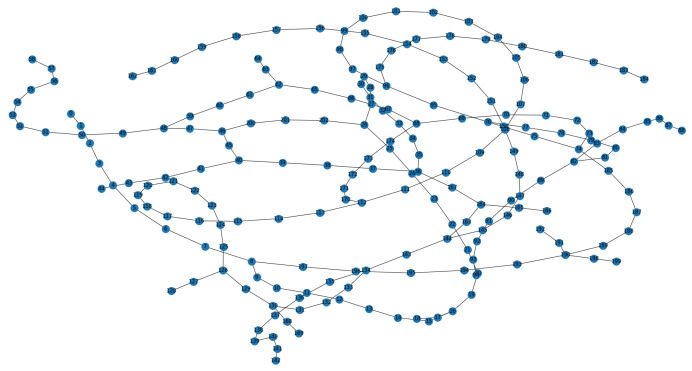
Experience node topology graph for maze environment.

**Figure 15:**
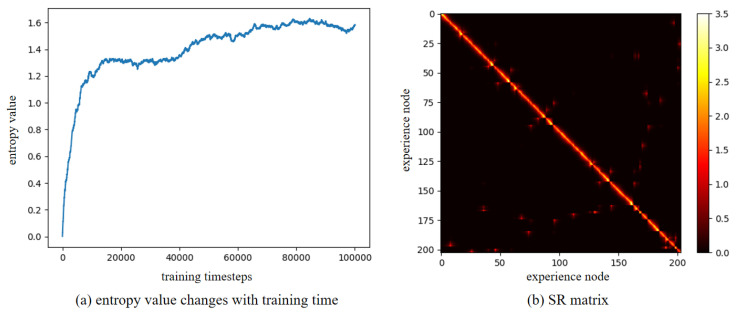
The training results for the maze environment.

Furthermore, to verify the robustness of our method in a dynamic environment, the maze experiments were conducted. We first construct a maze environment, with its layout shown in Figure 13a. In this maze, the unoccupied areas are passable. Then, the Depth-First Search algorithm is utilized to enable the agent to fully explore the maze environment and return to the starting point. In Figure 13a, the red dots represent experience nodes, which are uniformly distributed in these unoccupied areas. Figure 13b shows the experience node generation and recall performance; the agent completes the exploration in 413 timesteps, and generates a total of 203 experience nodes. Due to the characteristics of the maze layout, path backtracking is inevitable. Based on the connectivity of the experience nodes, an experience node topology graph is constructed, as shown in Figure 14. The connections between experience nodes indicate path traversability. Based on the experience node topology graph, the SR model is trained for 100,000 timesteps. Figure 15a demonstrates the changes in information entropy value with training timesteps; it can be seen that the SR model has converged after approximately 50,000 timesteps. The heatmap of the SR matrix is displayed in Figure 15b.

**Figure 16:**
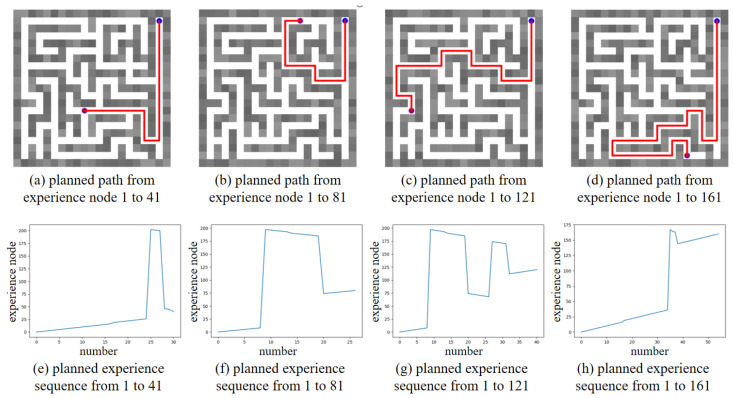
The global path planning results in unchanged maze environment. The blue dot represents the start point, the purple dot represents the goal point, and the red line in maze represents the planned path.

Based on the converged SR matrix, several global path planning experiments were conducted. Figure 16 shows the global path planning results, with the planned paths to different goals illustrated in Figure 16a–d, and the corresponding planned experience sequences shown in Figure 16e–h. The results clearly demonstrate that our method can reorganize the sequence of experience nodes and plan an optimal global path to the goal, where the optimal path consists of the minimum number of experience nodes. Additionally, we compared the planning time consumption between our method and the Dijkstra method, as shown in Table 4. The comparison highlights that our method achieves higher planning efficiency compared to the Dijkstra method.

**Figure 17:**
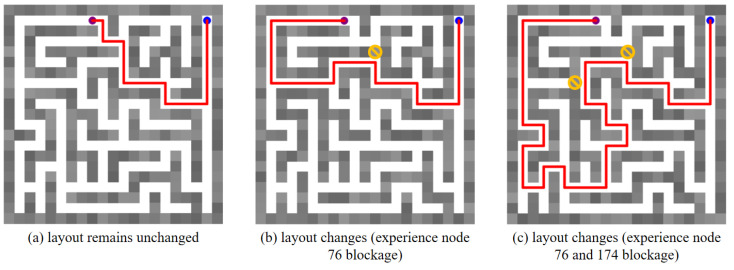
The global path planning results in a changed maze environment. The blue dot represents the start point, the purple dot represents the goal point, the red line in the maze indicates the planned path, and the yellow prohibitory mark represents an obstacle.

Moreover, we place obstacles in the maze environment, which block the previously traversable paths. Under such circumstances, the robot’s global planning ability is verified. Figure 17 describes the global path planning results in the changed maze environment, in which the blue dot represents the start point (experience node 1), the purple dot represents the goal point (experience node 100), the red line indicates the planned global path, and the yellow prohibitory mark represents the placed obstacle. Figure 17a shows the planned global path from experience node 1 to experience node 100. In Figure 17b, an obstacle has been placed at experience node 76, preventing the agent from reaching the goal along the previously planned path. Consequently, when the robot encounters a blockage, it can select its next experience node directly based on the SR matrix without the need to retrain the SR model. Similarly, two obstacles are placed at experience nodes 76 and 174 in Figure 17c. It is evident that the agent successfully plans an optimal global path to the goal despite these obstacles.

## 5. Conclusions

This work presents an innovative bionic goal-oriented path planning approach for mobile robots. By utilizing a spatial experience map, we construct a successor representation model through reinforcement learning, and develop a goal-oriented predictive map to quickly anticipate the experience sequence of the navigation goal. This novel approach enables mobile robots to rapidly plan the optimal global path. The proposed method was evaluated against the traditional Dijkstra path planning method. The experimental results demonstrate that our approach exhibits improved adaptability to environmental changes and efficiently plans the optimal path.

## 6. Discussion

This work contributes a new perspective to mobile robot global path planning. The bionic approach enhances the performance of mobile robots in dynamic and complex environments, while also paving the way for future research in this field. Unlike most bio-inspired navigation methods that primarily emphasize biological plausibility, our proposed bionic goal-directed approach achieves the dual optimization of both biological plausibility and practical applicability. Moreover, our method achieves robustness comparable to classical Dijkstra path planning method while guaranteeing path optimality and demonstrating superior computational efficiency.

The proposed bionic goal-oriented path planning method may have certain limitations that deserve further exploration in future work.

- Environmental complexity: Our method may face challenges in highly unpredictable or rapidly changing environments, such as home service and factory surveillance scenarios. Future research could investigate adaptive mechanisms to enhance the robustness performance under such conditions.
- Parameter sensitivity: The effectiveness of our method can be influenced by specific parameter choices. We have included a preliminary sensitivity analysis to highlight how variations in key parameters affect performance, and we suggest that further analysis could provide deeper insights.
- Scalability: Although the current implementation performs well in simulated scenarios, scalability to larger and more complex environments remains an area for future exploration.
- System biological plausibility: More advanced biological inspiration mechanisms could enable more intelligent and autonomous navigation for mobile robots.

## Figures and Tables

**Table 1:** SR matrix building algorithm.

Reinforcement Learning-Based SR Matrix Building Algorithm
Input: Experience map
Output: SR matrix
(1) Obtain the robotic state nodes according to the constructed experience map
(2) Obtain the adjacent selectable state nodes for each state node
(3) Initialize the robot’s current state as the initial state, initialize the training parameters of SR model
(4) Set the policy as a random policy, and initialize the SR matrix as an identity matrix
(5) Obtain the adjacent nodes of the robot’s current node, and set the connection probabilities of these nodes to the current node to be the same
(6) For all state node in the experience map, calculate and update $\hat{T}$ for each state’s adjacent nodes, as well as update the connection probability $p_{ik}\left(t+1\right)$
(7) Obtain the updated SR matrix and convergence information entropy H(t)
(8) Determine whether the model is converged: if no, go to (6); if yes, output the SR matrix
(9) Obtain the trained SR matrix for the experience map

**Table 2:** The comparison of time cost for global path planning based on SynPerData.

Method	Experience (a)	Experience (b)	Experience (c)	Experience (d)
Dijkstra	1.106	1.411	0.932	1.117
Ours	0.289	0.472	0.226	0.770

The time unit is milliseconds (ms).

**Table 3:** The comparison of time cost for global path planning based on QUTCarparkData.

Method	Experience (a)	Experience (b)	Experience (c)	Experience (d)
Dijkstra	0.267	0.310	0.395	0.311
Ours	0.118	0.120	0.240	0.212

The time unit is milliseconds (ms).

**Table 4:** The comparison of time cost for global path planning for maze environment.

Method	Experience (a)	Experience (b)	Experience (c)	Experience (d)
Dijkstra	0.115	0.164	0.218	0.216
Ours	0.061	0.075	0.105	0.108

The time unit is milliseconds (ms).

## Data Availability

The data that support the findings of this study are available from the corresponding author upon reasonable request.
